# In Vitro Degradation Studies of 3D-Printed Thermoplastic Polyurethane for the Design of Vascular Implant

**DOI:** 10.3390/ma18214948

**Published:** 2025-10-29

**Authors:** Kim Vanden Broeck, Marie-Stella M’Bengue, Thomas Mesnard, Mickaël Maton, Nicolas Tabary, Jonathan Sobocinski, Bernard Martel, Nicolas Blanchemain

**Affiliations:** 1 University of Lille, INSERM, CHU Lille, U1008, F-59000 Lille, France; kim.vandenbroeck@univ-lille.fr (K.V.B.); mariestella.mbengue@gmail.com (M.-S.M.); thomas.mesnard@univ-lille.fr (T.M.); mickael.maton@univ-lille.fr (M.M.); jonathan.sobocinski@univ-lille.fr (J.S.); 2 University of Lille, CNRS, INRAE, Centrale Lille, UMR 8207—UMET—Unité Matériaux et Transformations, F-59000 Lille, France; nicolas.tabary@univ-lille.fr (N.T.); bernard.martel@univ-lille.fr (B.M.); 3 Institut Coeur Poumon, Regional Hospital Center University of Lille (CHRU Lille), 2 Avenue Oscar Lambret, F-59000 Lille, France

**Keywords:** 3D printing, thermoplastic polyurethane, vascular implant, in vitro ageing, material degradation, surface properties, mechanical properties

## Abstract

Three-dimensional printing has emerged as a promising technology in endovascular surgery for the production of patient-specific stent-grafts. Thermoplastic polyurethane (TPU) is widely used for this purpose due to its favourable biocompatibility, hemocompatibility, and mechanical properties. However, its long-term stability under physiological conditions remains uncertain. This study evaluates the ageing behaviour of 3D-printed TPU stent-grafts under accelerated oxidative conditions (20% H_2_O_2_–0.1 M CoCl_2_) over three months, corresponding to approximately 45 months in vivo, and during three months in hydrolytic (0.1 M NaOH) conditions. Mechanical, physicochemical, thermal, and surface properties were periodically analysed. Differential scanning calorimetry revealed a decrease in crystallisation enthalpy of 41% and a reduction in melting enthalpy of 29% after hydrolytic ageing, whereas no decrease was observed after oxidative ageing. Despite these chemical changes, size exclusion chromatography indicated minimal chain scission. However, spectroscopy and microscopy showed minor chain scission and additive migration (antioxidant and lubricant). Nevertheless, tensile testing highlighted that mechanical performance remained within clinically acceptable ranges. These findings demonstrate that 3D-printed TPU vascular implants retain essential properties under prolonged simulated ageing, supporting their safety and durability for vascular applications.

## 1. Introduction

Thermoplastic polyurethanes (TPUs) are versatile polymers widely employed in industrial applications, including coatings, adhesives, and materials engineering, as well as in the biomedical field [1,2,3,4]. TPU are macromolecules composed of two distinct segments. The first are aliphatic polyether or polyester segments that constitute the soft segments (SS), and the second are aliphatic or aromatic polyurethane chains formed by a diisocyanate and a chain extender, which constitute the hard segments (HS) [5]. These two types of segments lead to the formation of a biphasic material with crystalline clusters composed of HS held by hydrogen bonds, π-π stacking, and amorphous clusters with aliphatic polyols [6]. Upon heating, hydrogen bonds dissociate due to increased molecular mobility, enabling dispersion of HS within the amorphous phase. Cooling reverses this process, restoring hydrogen bonding and thereby conferring the thermoplastic properties of TPU [6,7]. Owing to their biocompatibility and excellent hemocompatibility, TPUs are widely used in the design of implantable and non-implantable cardiovascular devices, including blood bags, catheters, and vascular prostheses [2,8,9]. Among polyurethane families, poly(ether-urethane) has become the most prevalent due to its superior resistance to hydrolytic degradation compared with poly(ester-urethane) [10,11,12].

Three-dimensional (3D) printing enables the fabrication of physical objects from digital models through layer-by-layer material deposition. Originally developed for aeronautical, aerospace, automotive, and sports industries, 3D printing is increasingly applied to the production of patient-specific implantable medical devices, particularly in vascular, dental, and orthopaedic applications [13,14,15,16]. One of the most widely used techniques, fused deposition modelling (FDM), relies on the extrusion of a thermoplastic filament deposited in molten form onto a heated substrate. Nevertheless, TPU and TPU-based blends have already been explored for 3D printing in several studies in the automotive, electrical, sensing, and shape-memory fields, showing promising results [17,18,19]. In the medical filed, polymers such as polycaprolactone and polylactic acid are already use to print stent by the FDM technique [13,20,21]. Although TPU is still limited in biomedical applications, 3D printing of polyurethane is emerging. For instance, Jung et al. developed a bio-based, shape-memory TPU filament for 3D/4D printing [5], and several studies have demonstrated the feasibility of producing TPU-based vascular stents via FDM, highlighting its potential in endovascular surgery [22,23]. Conventionally, endovascular stent-grafts consist of metallic stents covered with a synthetic textile (e.g., polyethylene terephthalate, PET) sewn onto the scaffold. In certain cases, patient-specific stent-grafts are required. However, the manufacturing time of this type of stent-graft is relatively long, typically ranging from 6 to 8 weeks. To shorten this delay and enable treatment in emergency situations, 3D printing can be used to produce the prosthesis in a reduced time. For clinical use, these devices must also be crimped into delivery catheters of ≤24 French (Fr) for abdominal aortic aneurysms [24] and ≤25 Fr for thoracic aortic aneurysms [25], which represents an additional constraint in the design and fabrication process.

In our recent work, we investigated the impact of filament extrusion, 3D printing, and sterilisation on TPU [18]. Although chain scission and additive migration occurred during processing, the mechanical properties remained unchanged. Following gamma sterilisation, the FDM-printed samples exhibited no haemolytic or cytotoxic effects and demonstrated reduced platelet adhesion and activation. Building on these findings, we patented the design of an endovascular graft consisting of a metallic stent covered by a 3D-printed TPU membrane (WO2024213841) [26]. Given that TPU stability varies with composition and HS/SS ratio, it is essential to evaluate the effects of ageing on its physicochemical, mechanical, and biological properties [10,27,28,29].

In biological environments, polyurethane degradation primarily results from hydrolysis, oxidative stress, enzymatic activity, and mechanical forces [8,10]. Following implantation, macrophages and foreign-body giant cells adhere to the material surface, releasing reactive oxygen species (ROS) that trigger oxidative degradation through chain scission and/or crosslinking [30,31]. Hydrolytic degradation arises under conditions of high moisture, leading to cleavage of urethane linkages [32].

Previous studies have employed accelerated ageing models to investigate these processes. For example, Christenson et al. demonstrated that a 20% H_2_O_2_–0.1 M CoCl_2_ (H_2_O_2_/CoCl_2_) solution reliably simulates in vivo oxidative ageing via hydroxyl radical production through the Haber–Weiss reaction [28,33]. They also showed that ageing of TPU for 24 days in solution reproduced the degradation observed after one year of in vivo implantation, as evidenced by morphological changes detected with SEM and by chemical modifications revealed by ATR-FTIR analysis [28,30,34]. Hydrolytic degradation, on the other hand, is often simulated using phosphate-buffered saline at temperatures ranging from 37 to 85 °C or NaOH solutions [30,35,36]. However, to date, the long-term effects of ageing on 3D-printed TPU have not been investigated.

The present study investigates the accelerated ageing and comprehensive evaluation of a 3D-printed TPU vascular device. To mimic in vivo conditions, oxidative and hydrolytic ageing environments were established in vitro. Mechanical, physicochemical, thermal, and surface properties of the material were monitored over time to assess long-term stability. Accelerated ageing protocols in an oxidative solution were carried out over 3 months (91 days), which is equivalent to 45 months of in vivo exposure (with 24 days corresponding to 1 year or 12 months [28,30,34]). This provided critical insights into the durability and performance of 3D-printed TPU-based vascular devices.

## 2. Materials and Methods

### 2.1. Materials

Medical-grade TPU, Elastollan^®^ 1185A supplied by BASF (Lemförde, Germany), was received in raw pellet form. TPU is polyether-based with polytetramethylene oxide (PTMO) as SS, 4,4-diphenylmethane diisocyanate (4,4-MDI) as HS, and 1,4-butane diol (1,4-BDO) as a chain extender. Tetrahydrofuran (THF) with HPLC grade (≥99.8%) was supplied by Honeywell Riedel-de Haën^TM^ (Seelze, Germany). Sodium hydroxide pellets (≥97%) were supplied by Fisher Scientific (Geel, Belgium). A solution of 50% hydrogen peroxide was supplied by VWR (Fontenay-sous-Bois, France). Cobalt (II) chloride anhydrous was provided by ThermoFisher (Kandel, Germany).

### 2.2. Sample Preparation

#### 2.2.1. Three-Dimensional Printing by Fused Modelling Deposition (FDM)

Three-dimensional-printed tubular samples were fabricated, as described in a previous study [18]. Briefly, the raw pellets were extruded (Composer 350, 3devo, Utrecht, The Netherlands) into a 1.75 mm diameter filament with extrusion temperatures from the feeding to nozzle of 155 °C, 175 °C, 181 °C, and 185 °C according to the fabricant recommendations (BASF, Lemförde, Germany). The rotation speed of the single extrusion screw was 2.8 rpm. Tubular samples (height: 75 mm, diameter: 11 and 12 mm, wall thickness: 0.25 mm) were designed with open-source software (On Shape v1.205, Boston, MA, USA), sliced (Simplify3D-4.1.2, Cincinnati, OH, USA), and printed by FDM (Stream 20 Dual MK2, Volumic, Nice, France).

#### 2.2.2. Manufacture of Prosthesis Prototype

**Figure 1:**
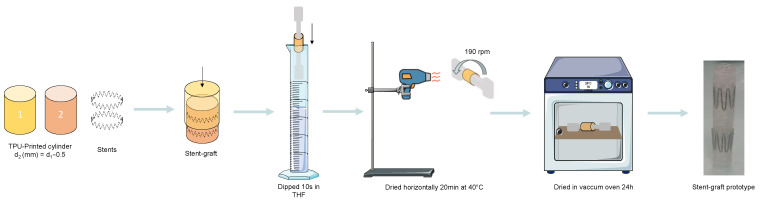
Manufacturing process of the 3D-printed endovascular stent-graft.

Two cylinders with a diameter difference of 0.5 mm were assembled. To achieve this, the Z stent was inserted into the outer cylinder before adding the inner cylinder. The stent-graft was then dipped in a THF solution for 10 s to physically bond the two membranes. Samples were then air-dried horizontally using a rotary mandrel (190 RPM, IKA^®^ RW 20 digital, Fisher scientific, Waltham, MA, USA) and a heat gun (40 °C, Steinel HL 1920 E, Conrad, Hirschau, Germany) for 20 min. Prototypes were then placed in a vacuum oven at 70 cmHg for 24 h to allow the complete evaporation of the solvent as described in the patent WO2024213841 [26] (Figure 1).

#### 2.2.3. Sterilisation

The stent-graft prototypes were sterilised using gamma (γ) irradiation at a dose of 40 kGy, in accordance with the ISO 11137-2:2013 [37]. This dosage ensures a microbial survival probability of less than or equal to 10^−6^.

### 2.3. Sample Ageing

The ageing study was carried out using ISO 10993-13:2010 [38]. Ageing was followed in hydrolytic and in oxidative solutions. A hydrolytic solution, 0.1 M NaOH, was obtained by the dissolution of sodium hydroxide pellets in ultrapure water under stirring and was stored at 4 °C. The oxidative solution, 20% H_2_O_2_ + 0.1 M CoCl_2_, was obtained by dissolving cobalt chloride in ultrapure water, followed by the addition of the 50% H_2_O_2_ solution. This solution allows the production of hydroxyl radical through the Haber–Weiss reaction [28,33]:

Co^2+^ + H_2_O_2_ → Co^3+^ + HO^−^ + HO^·^

Samples (height: 75 mm, diameter: 12 mm) were immersed in 35 mL solutions and incubated at 37 °C under agitation at 80 rpm. Each solution was renewed every 3 days. Samples (*n* = 3) were removed at 2 weeks, 1 month, and 3 months. Samples immersed in 20% H_2_O_2_–0.1 M CoCl_2_ are named H_2_O_2_/CoCl_2_-2w, H_2_O_2_/CoCl_2_-1m, and H_2_O_2_/CoCl_2_-3m and, for samples immersed in 0.1 M of NaOH, NaOH-2w, NaOH-1m, and NaOH-3m for the above-mentioned sampling times. The collected samples were rinsed three times with ultrapure water and dried for 24 h in a vacuum oven OV-11 (Fisher Scientific, Waltham, MA, USA) at 25 °C and 70 cmHg.

### 2.4. Characterisation of Samples

#### 2.4.1. Size Exclusion Chromatography (SEC)

Size exclusion chromatography (SEC) was performed using a WATERS E2695 chromatograph (Waters Corporation, Milford, MA, USA) equipped with Styragel HR-1, HR-3, and HR-4 columns (molecular weight range: 500–600,000 g.mol^−1^, size: 7.8 mm × 300 mm) and coupled with a differential refractometer (Optilab^®^-T-rEX, Wyatt Technology, Santa Barbara, CA, USA). The system was calibrated using polystyrene standards. A total of 15 mg of the TPU sample was dissolved in 3 mL of tetrahydrofuran (THF) (Honeywell, Charlotte, NC, USA); 1 mL of toluene (VWR, Fontenay-sous-bois, France) was added per litre of THF as a flow marker. The resulting solutions were filtered through a 0.45 µm PTFE membrane and transferred into glass vials. The samples were eluted with a mobile phase consisting of THF at a flow rate of 1 mL/min. The injection volume was 75 μL. Data processing was conducted using Astra 6 software (Wyatt Technology, Santa Barbara, USA) and the molar masses were expressed in polystyrene equivalent. Three samples per series (*n* = 3) were analysed. For each sample, measurements were taken in triplicate to verify within-sample homogeneity.

#### 2.4.2. Differential Scanning Calorimetry (DSC)

Differential scanning calorimetry was conducted using a DSC 300 Caliris^®^ Select (Netzsch, Pontault-Combault, France). Each sample was sealed in standard aluminium pans and analysed under an inert nitrogen atmosphere (20 mL.min^−1^ flow rate). An empty aluminium pan served as the reference. Heat flow thermograms were recorded over a temperature range from −70 °C to +270 °C, following a heating–cooling–heating cycle at a rate of 10 °C.min^−1^. The glass transition temperature (Tg) was determined as the midpoint of the transition, while melting temperature (Tm) and crystallisation temperature (Tc) were identified as the peak of the endothermic and exothermic events. All analyses were performed in triplicate.

#### 2.4.3. Infrared Spectroscopy (ATR-FTIR)

Fourier transform infrared (FTIR) spectroscopy was performed on the FDM samples using the attenuated total reflectance (ATR) technique. The analyses were conducted with a SpectrumTwo IR spectrometer (Perkin Elmer, Villebon-Sur-Yvette, France) and its associated software (Spectrum version 10.6.0). Measurements were carried out at room temperature on three independent samples (*n* = 3), with triplicate readings per sample, and recorded in absorbance mode. Each spectrum was obtained after 16 scans over a wavenumber range of 4000 cm^−1^ to 400 cm^−1^, with a resolution of 2 cm^−1^. The resulting data were exported in ASCII format and processed using Excel to plot the absorbance spectra. Spectra are normalised relative to the 1413 cm^−1^ band.

#### 2.4.4. Scanning Electron Microscope (SEM)

The surface of the samples was analysed using a FlexSEM1000II scanning electron microscope (Hitachi, Tokyo, Japan). Prior to imaging, a 100 Å layer of chromium was sputter-coated onto the samples to ensure conductivity with a Quorum Q150T ES metallizer (Quorum technology, Sacramento, CA, USA). Observations were carried out at an accelerating voltage of 5 kV and an emission current of 10 µA.

#### 2.4.5. Optical Profilometry

Surface roughness and 3D topography maps were obtained using a confocal technique with an S Neox surface profiler (Sensofar, Terrassa, Spain). Data acquisition and analysis were performed using Sensoview 2.4.1 software. Roughness parameters were calculated according to ISO 25178-2:2021 [39], applying an S-filter of 250 nm, a third-degree polynomial as the F-operator, and an L-filter of 8 µm. The calculated roughness corresponds to the average of measurements taken on three different samples.

#### 2.4.6. Mechanical Tests

Dumbbell test pieces (12 mm × 4 mm) were cut in the parallel and perpendicular directions to the printing layers of samples. The test bench used was a universal traction machine Autograph AGS-X (Shimadzu, Kyoto, Japan). The tensile tests were carried out with an initial strain rate of 3.5 × 10^−3^ s^−1^ until the rupture of the sample. The Young’s modulus, ultimate tensile strength, and elongation at break were determined from the tensile curve. The tests were carried out in triplicate and according to the ASTM D638 standard [40] relating to the tensile properties of plastics.

## 3. Results

The evolution of the prototype during the ageing was studied by immersion of the sample in the hydrolytic and oxidant media. Media were analysed after 2 weeks, 1 month, and 3 months of TPU degradation.

### 3.1. Evolution of Thermal Analysis

**Figure 2:**
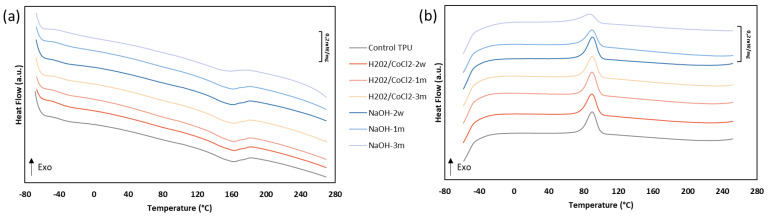
DSC from (**a**) the first cooling run and (**b**) second heating run for the TPU control, H_2_O_2_-CoCl_2_-2w, H_2_O_2_-CoCl_2_-1m, H_2_O_2_-CoCl_2_-3m, NaOH-2w, NaOH-1m, and NaOH-3m samples (*n* = 3).

The DSC of the first cooling and the second heating cycle of TPU and aged TPU are shown in Figure 2 and collected data from DSC are shown in Table 1. The first inflection point in the range of −40 °C to −45 °C is associated with the glass transition (Tg) of the soft domains of TPU. The exothermic transition in the range of 85 °C to 95 °C is associated with the crystallisation temperature of the HS, whereas the endothermic transition around 160 °C corresponds to the melting of those HS in the TPU [10,18,41]. Oxidative ageing does not reveal notable changes **in contrast to** hydrolysis ageing. In the hydrolysis condition, the enthalpy of crystallisation (ΔHc) decreased after 1 month of ageing and continues to decrease with a drop of 41% after 3 months. In addition, a decrease of 29% in the melting enthalpy (ΔHm) is also visible after these 3 months. These decreases are associated with an offset of melting and crystallisation temperature, which is particularly pronounced on Tm, with 161 ± 0.5 °C for TPU versus 150 ± 4.2 °C for the NaOH-3m sample. The chain scission during hydrolytic degradation occurs in HS at the level of urethane function, whereas it occurs in SS at the level of ether function during oxidative degradation [28,32,42]. These mechanisms could explain the decrease in melting and crystallisation enthalpies, which are much greater in hydrolytic media than in oxidative media.

### 3.2. Evolution of Molecular Weights

**Figure 3:**
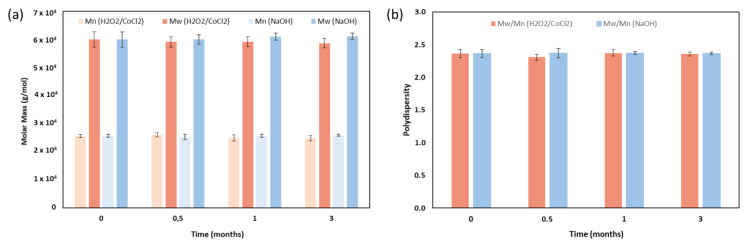
Impact of ageing under accelerated conditions on (**a**) Mw and Mn and (**b**) Ð. Data are expressed as mean ± SD (*n* = 3). Molar masses are expressed in polystyrene equivalent.

The occurrence of major chain scission was assessed by SEC analysis to monitor the evolution of molecular weights (Mw and Mn) and the polydispersity index (Ð) of the TPU during ageing. Over the 3-month ageing period, the molecular weights and Ð remained stable in both media (Figure 3). All samples exhibited molecular weights of around 25,000 g/mol for Mn and 60,000 g/mol for Mw, with a consistent Ð of approximately 2.36 (Table 2).

### 3.3. Evolution of Surface Properties

**Figure 4:**
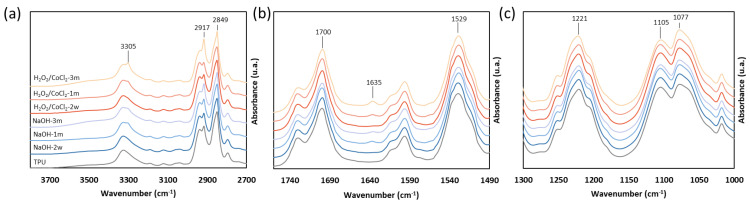
ATR-FTIR spectra zoom of TPU and aged samples from (**a**) 2700 to 3800 cm^−1^, (**b**) 1490 and 1760 cm^−1^, and (**c**) 100 ad 1300 cm^−1^ (*n* = 3).

FTIR analysis was carried out to monitor surface functional groups during ageing to better understand the decrease in thermal properties in a hydrolytic environment. The complete spectra of the TPU and aged TPU, visible in Figure S1 (Supplementary Materials), highlighted characteristic peaks such as the N-H stretching vibration at 3320 cm^−1^, a strong C=O stretching vibration at 1700 cm^−1^ characteristic of the urethane carbonyl group, and the ether peak of the soft segment at 1110 cm^−1^. After ageing, spectra show some differences between unaged and aged TPU (Figure 4). For all ageing, shoulders progressively increase during ageing at 2917 cm^−1^, 2849 cm^−1^, 3305 cm^−1^, and 1635 cm^−1^ corresponding to a -CH_2_ stretching vibration and a -NH stretching and deformation vibration (Figure 4a,b). Mrad et al. showed that peaks at 2848 cm^−1^, 2916 cm^−1^, and 3307 cm^−1^ are characteristic of the presence of ethylene-bis-stearamid (EBS), a lubricant commonly used to facilitate polyurethane extrusion [43,44]. Nouman et al. also showed that the emergence of a peak at 1634 cm^−1^ can also be attributed to the migration of EBS, in addition to the first three peaks [45]. This new peak at 1635 cm^−1^ can also be attributed to the presence of an aromatic amine caused by a hard segment chain scission on the surface of TPU [28,46].

After 3 months of ageing in both conditions, a new broad band with low intensity appeared at 3650 cm^−1^, corresponding to an alcohol or a phenolic alcohol stretching vibration. This peak can be attributed to a phenolic antioxidant additive or a chain scission. Nouman et al. have identified the blooming of phenolic antioxidants such as Irganox^®^ in TPU catheters [45,47,48]. For hydrolysis ageing, a slight decrease in the 1700, 1529, 1221, and 1077 cm^−1^ peaks can be observed after 3 months of ageing (Figure 4b,c). These peaks are characteristic of the carbamate bond with the C=O stretching vibration for 1700 cm^−1^ peak, the C-N stretching and N-H deformation for the 1529 cm^−1^ peak, the C-O stretching vibration of the ester urethane (-N-CO-O-) for the 1221 cm^−1^ peak, and the C-O stretching vibration of the urethane (C-O-C) is attributed to the 1077 cm^−1^ peak [49,50]. The slight decrease in these peaks can highlight a minimal split in the urethane function. On the other hand, a decrease in the 1105 cm^−1^ peak, corresponding to the C-O stretching vibration in the ether function, is visible after 3 months of oxidative ageing.

The FTIR analysis highlighted additives migration, with the identification of EBS and a phenolic antioxidant on the surface of the TPU. Moreover, the slit decrease in the urethane characteristic peaks after the 3 months of ageing in the hydrolysis condition and the ether peak in the oxidative condition seems to confirm small-scale degradation.

**Figure 5:**
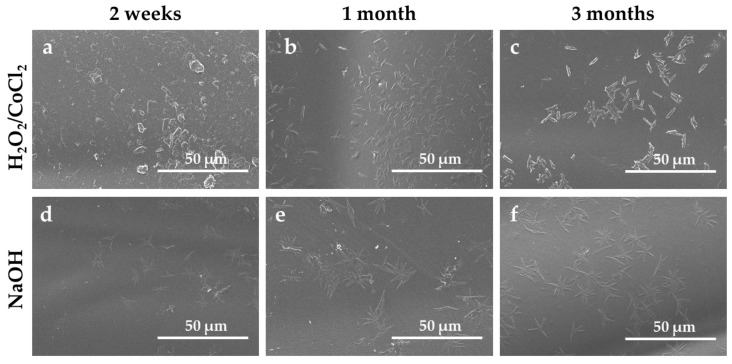
SEM images of TPU surfaces after (**a**–**c**) oxidative and (**d**–**f**) hydrolytic ageing under accelerated condition.

The SEM images confirmed additive migration on the surface with the presence of two types of aggregates (Figure 5). After two weeks of ageing, the emergence of some aggregates with a circular shape and needle shape is visible on the TPU surface in both conditions. The needle-shaped aggregates are characteristic, according to Nouman et al., of phenolic antioxidant such as Irganox^®^ [45]. Figure 5 shows that the antioxidant aggregate increases with the time of ageing in both conditions. Furthermore, the circular-shaped aggregates, corresponding to the EBS lubricant, also increase with ageing time (Figure S2). These observations are according to the FTIR results, which showed a growing intensity of the characteristic EBS band, potentially due to additive migration and the emergence of alcohol or phenol-related bands. Such additive blooming not only modifies the chemical composition of the surface but also increases roughness, which has been correlated with platelet adhesion and thrombus formation in vascular devices [51,52].

**Figure 6:**
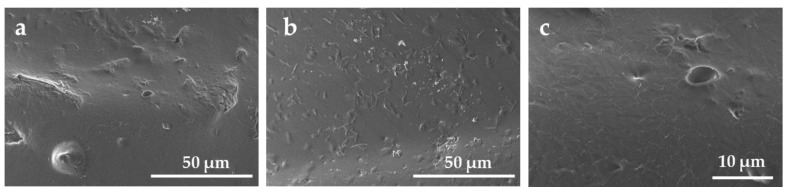
SEM images focus on the microcracking, micro-holes (**a**,**c**) and pitting (**b**,**c**) on the surface of the H_2_O_2_/CoCl_2_-3m sample.

After three months of oxidative ageing, some small pits (Figure 6b,c), microcracking, and holes (Figure 6a,c) appeared in certain areas on the surface of the sample. Indeed, polyurethane ethers are particularly known for their susceptibility to oxidative degradation, which can significantly contribute to surface embrittlement and stress cracking over time [10]. Christenson et al. have already observed pitting of the surface and had attributed this to the extraction of low-molecular-weight degradation products caused by a chain scission [30,46]. The presence of antioxidants mitigates these phenomena. However, their potential migration may reduce their concentration at the surface, thereby increasing its susceptibility to oxidation.

**Figure 7:**
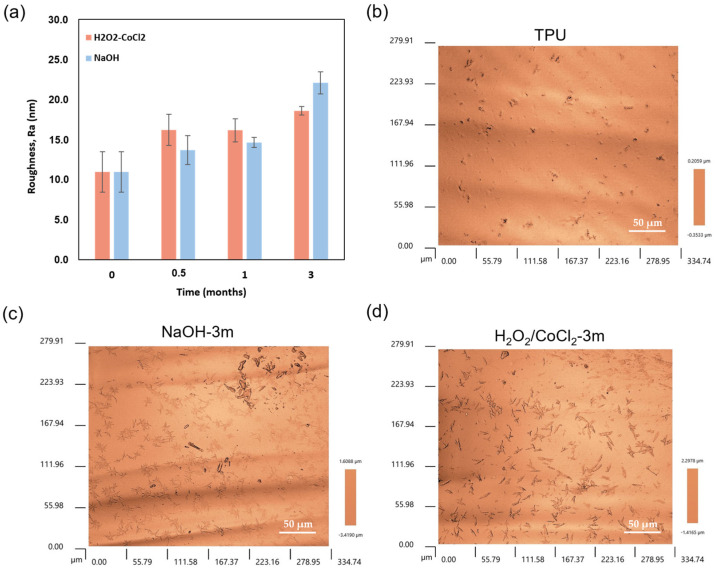
(**a**) Evolution of the roughness of aged samples (*n* = 3) and (**b**) 3D surface topography map of TPU, (**c**) NaOH-3m, and (**d**) H_2_O_2_/CoCl_2_-3m samples.

To control the impact of additive migration and stress cracking over time, surface roughness was monitored during the process (Figure 7a). An increase in surface roughness (Ra) was observed under both conditions. To verify the implication of additive migration and stress cracking of this increase in roughness, 3D surface topography maps are made (Figure 7b–d). These maps provide evidence of the implication of the additive migration, with the presence of aggregates on the surface, contrary to the pitting or micro-holes. More specifically, the presence of a large number of needle-shaped aggregates, associated with the antioxidant additive, appears to be the primary cause of this evolution. However, the effect was more pronounced under hydrolytic conditions, where Ra increased from 11.0 ± 2.5 nm to 22.1 ± 1.4 nm, compared to oxidative conditions, where it reached 18.6 ± 0.5 nm (Table 3). The chain scission occurring at the urethane function, located in the hard phase and revealed by FTIR analysis under hydrolytic conditions, could contribute to enhanced additive migration.

**Figure 8:**
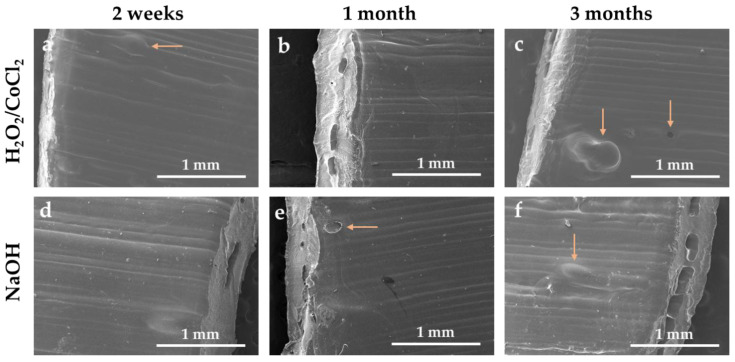
SEM images of TPU interface after (**a**–**c**) oxidative and (**e**–**f**) hydrolytic ageing.

As explained in Section 2, the prostheses prototypes consist of one TPU cylinder fitted inside another and welded by rapid immersion in THF and drying. The SEM images of the TPU showed some residual interlayer air pockets trapped between the internal and external cylinders visible by white arrows on Figure S3 (Supplementary Materials). SEM analysis was performed to monitor the evolution of these air pockets. After immersion of the sample in both ageing solutions, these air pockets increased drastically and caused the emergence of holes and air bubbles on the sample surfaces (orange arrows in Figure 8), which can lead to the separation of both cylinders and to the embrittlement of the prototype vascular graft.

### 3.4. Evolution of Mechanical Properties

**Figure 9:**
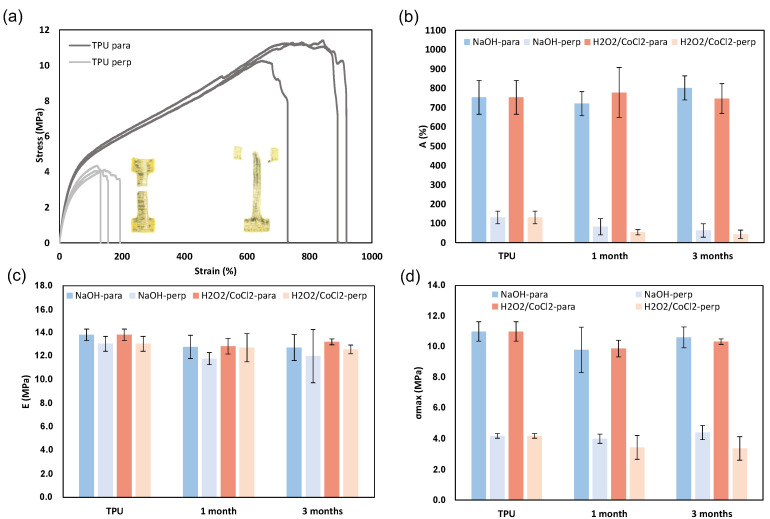
Impact of ageing under accelerated conditions on the mechanical properties of 3D-printed samples (*n* = 3): (**a**) tensile curve of TPU, (**b**) Young’s modulus, (**c**) ultimate tensile strength, (**d**) and elongation at break (mean ± SD). Inserted in (**a**), the photographs of samples after tensile testing in parallel and perpendicular orientations.

Tensile tests were carried out (Figure 9a, Table 4) to assess the impact of ageing on the mechanical properties. These tests were conducted in both parallel and perpendicular directions relative to the printing direction [18]. The prototype displayed elastomeric behaviour, with a Young’s modulus of 13.8 ± 0.5 MPa and 12.8 ± 0.5 MPa in the parallel and perpendicular directions, respectively. The variation in ultimate tensile strength and elongation at break between the perpendicular and parallel directions arises from weaker interlayer bonding in the perpendicular orientation, forming stress concentrations that serve as initiation and propagation sites for failure. In all samples, regardless of orientation, failure occurred through delamination along the printed layers, confirming that the weak zone is located at the interface of both cylinders (Figure 9a). Under accelerated ageing conditions in parallel orientation, the Young’s modulus, elongation at break, and ultimate tensile strength remained stable over a period of three months, with values of 12.8 ± 1.1 MPa, 801 ± 62%, and 10.6 ± 0.7 MPa, respectively, for the NaOH aged 3 months (Figure 9b–d). However, in the perpendicular orientation, a decrease in the elongation at break is highlighted after 3 months of ageing in oxidative and hydrolysis conditions, with a value of 43 ± 22% and 63 ± 35%, respectively, versus 130 ± 33% for TPU. The absence of any change in mechanical properties along the parallel direction suggests that the reduction in crystallinity had no significant effect. In contrast, the presence of air pockets between both fitted cylinders appears to influence the strain at break in the perpendicular direction, likely due to delamination.

## 4. Discussion

Our study aimed to evaluate the in vitro degradation of a 3D-printed medical-grade thermoplastic polyurethane. Degradation studies were carried out under accelerated conditions. According to Christenson et al., ageing of TPU for 24 days in an oxidative solution reproduced the degradation observed after one year of in vivo implantation, as evidenced by morphological changes detected with SEM and by chemical modifications revealed in ATR-FTIR spectra [28,30,34]. In this study, the long-term behaviour of TPU was assessed for up to 3 years and 9 months in an oxidative solution. Additionally, the behaviour of TPU was monitored in an accelerated hydrolytic solution over 3 months. To the best of our knowledge, despite the use of 0.1 M NaOH for the accelerated condition, no equivalence between in vitro and in vivo degradation rates has been established in the literature [35].

**Figure 10:**
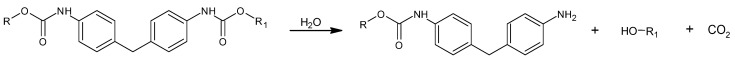
Hydrolysis of carbamate function in a poly(ether-urethane). Based on [10,36].

After ageing under hydrolytic degradation in 0.1 M NaOH solution, SEC analysis showed no significant decrease in molar mass, indicating that no major chain scission occurred. However, the thermal analysis performed by DSC highlights a decrease in crystallisation and melting enthalpies of 41% and 29%, respectively, which only occurs under the hydrolytic condition. This observation was correlated with ATR-FTIR spectra, which show a decrease in the urethane characteristic peaks and the emergence of an alcohol peak, probably caused by minor chain scission occurring on the surface. Indeed, the hydrolysis of the urethane function caused the presence of alcohol, amine, and carbon dioxide (Figure 10) [36]. This urethane chain scission, which is located in the crystallised phase, leads to smaller crystals and so can reduce TPU crystallinity.

**Figure 11:**
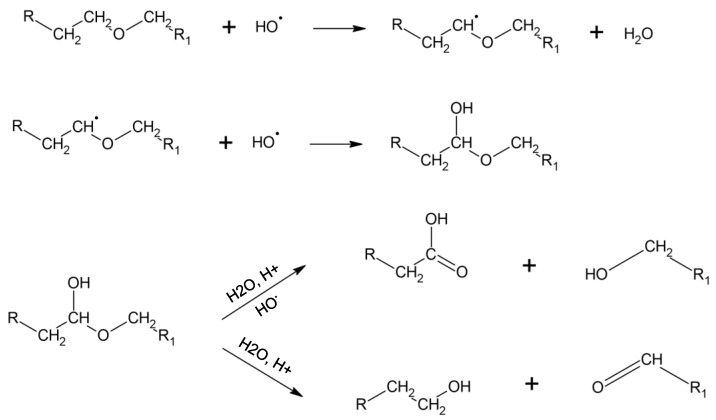
Soft segment oxidation in a poly(ether-urethane). Based on [10,30].

On the other hand, under oxidative conditions, no significant decrease in crystallinity was observed. This stability may be related to the specific nature of the degradation mechanism. Hydroxyl radicals, generated via the Haber–Weiss reaction to mimic the reactive oxygen species (ROS) released by adherent cells in vivo, are known to induce chain scission in both ether and urethane linkages. Xie et al. reported that ether bonds are more susceptible to oxidative degradation than urethane groups [10]. FTIR spectra confirm the presence of a slight decrease in the ether peaks (1105 cm^−1^) and an increase in the alcohol peaks (3200–3600 cm^−1^), visible after 3 months of ageing. This observation was correlated with the degradation mechanism, which primarily occurs by the attack of the hydroxyl radical on the polyether α-methylene hydrogen atom to form aldehyde or carboxylic acid and alcohol (Figure 11) [10,46]. The SEM analysis showed the presence of stress cracking, with the emergence of pitting, cracking, and micro-holes. Pitting of the surface can be attributed to the extraction of low-molecular-weight degradation products caused by a chain scission [30,46]. This stress cracking is limited by the presence of antioxidants. However, their potential migration within the material may lead to a decreased concentration, thereby increasing the surface’s susceptibility to oxidation.

Additive migration was also highlighted during the ageing process in both conditions. The FTIR spectra showed the progressive emergence of characteristic peaks (3305, 2917, 2849, and 1635 cm^−1^) of a lubricant, the ethylene-bis-stearamid, and a possible phenolic antioxidant (board peak 3200-3600 cm^−1^) [28,43,44,45,46]. This observation can be attributed to additive migration, which may be explained by the surface chain scission. This is confirmed by the SEM analysis of the aged sample, which reveals the appearance of two types of aggregates corresponding to EBS, a lubricant, and phenolic antioxidant such as Irganox^®^. Indeed, previous studies in the literature have identified EBS and Irganox^®^ migration after the ageing of TPU catheters [44] and the needle-shaped aggregates are identified to be characteristic of Irganox^®^ [45,47,48]. Surface deterioration, particularly additive migration, is an important parameter, as these phenomena generate significant roughness that could lead to the thrombosis of the vascular device. Indeed, Linneweber et al. demonstrated that the surface roughness of a medical device not only affects the coagulation system but also the platelet adhesion [53]. They also showed that increasing the surface roughness from 50 nm to 200 nm and 400 nm led to an increase in platelet adhesion of approximately 40% and 76%, respectively, highlighting the critical role of surface topography in modulating thrombogenicity. Similarly, a study of Jayaraman et al. highlighted that the higher the roughness, the greater the risk of thrombosis [51]. To control that, roughness was monitored and showed an increase during ageing from 11.0 ± 2.5 nm to 22.1 ± 1.4 nm in hydrolytic conditions attributed to the antioxidant, with the presence of a large number of needle-shaped aggregates on the 3D surface topography maps. Moreover, the exudation of additives such as Irganox^®^ has been reported to negatively impact cytocompatibility of polyurethane catheters [45,47,48]. This suggests that additive migration in 3D-printed vascular devices could compromise long-term biocompatibility.

Finally, SEM observation showed surface degradation with the appearance of stress cracking and the increase in interlayer air pockets trapped between both fitted cylinders. Such formation of air pockets could generate a delamination of the double-layered graft and cause an early rupture, which is visible with the decrease in the elongation at break in the perpendicular orientation. However, the device would primarily be subjected to mechanical stresses in the parallel direction, under pulsatile arterial pressure, and is therefore not significantly affected by the slight decrease in perpendicular orientation. Moreover, all the other mechanical properties remained stable after the three months of ageing. Furthermore, a simulation performed by Li and Kleinstreuer showed that the maximal wall stress on the stent-graft reaches 1.76 MPa, which is largely lower than the ultimate tensile strength of the prototype before and after the ageing [51]. Thus, after this ageing process, the mechanical properties showed a slight deterioration but remained sufficient to guarantee the performance of the stent-graft.

## 5. Conclusions

Endovascular grafts were built by the assembly of 3D-printed TPU cylinders fitted one in another and bonded together by rapid immersion/drying steps in THF. This in vitro accelerated ageing study demonstrated the stability of the prototypes under oxidative conditions estimated to correspond to a period of 3 years and 9 months and under accelerated hydrolytic condition during 3 months. If SEC analysis could not detect any chain scissions, FTIR displayed small changes in bands associated with urethane functions, suggesting minor chain degradations. DSC also displayed limited evolution of the microstructure, with a decrease in melting and crystallisation enthalpies and a shift in crystallisation temperature. These degradations occurred through the cleavage of carbamate bonds under hydrolytic conditions and oxidation of ether groups under oxidative conditions. Such minor chemical alterations led to surface damage, including stress cracking and additive migration (antioxidant and lubricant present in the raw TPU). Ageing also provoked an extension of interstitial air pockets between the inner and the outer TPU cylinders of our prototypes. This presence of interlayer air pockets resulted in early rupture and a slight decrease in strain at break in the perpendicular orientation, whereas all other mechanical properties remained stable. Importantly, the mechanical performance of the material remained largely sufficient for vascular stent-graft applications, both before and after ageing. However, additive migration increased the surface roughness of the 3D-printed device, which may promote thrombus formation. As thrombosis is one of the most common complications after vascular device implantation, the risk may be further increased with 3D-printed devices. A study is currently underway to add an antithrombotic coating to the stent-graft. These results represent a proof of concept for the durability of 3D-printed TPU vascular prostheses. However, this study has some limitations, including the time of our ageing, too short for this type of vascular implant. Nevertheless, this study demonstrated a methodology based on several parallel analyses, such as molar mass measurement, thermal analysis, mechanical testing, and FTIR, which could be applied in long-term animal testing. This in vivo long-term ageing will be performed in future work, and the development of an antithrombotic coating to mitigate this risk will also be explored.

## Figures and Tables

**Table 1:** Collected DSC data (*n* = 3) from the first cooling and second heating run (mean ± SD). Tg, Tc, and Tm are the temperature of glass transition, crystallisation, and melting, respectively, while ΔHc and ΔHm are the enthalpy of crystallisation and melting, respectively.

	Tg (°C)	Tc (°C)	ΔHc (J/g)	Tm (°C)	ΔHm (J/g)
Control	−42 ± 0.9	90 ± 0.7	10.33 ± 0.35	161 ± 0.5	8.53 ± 0.39
H_2_O_2_/CoCl_2_-2w	−44 ± 0.8	93 ± 5.4	10.42 ± 0.25	161 ± 0.8	8.49 ± 0.19
H_2_O_2_/CoCl_2_-1m	−42 ± 0.9	90 ± 0.2	10.63 ± 0.37	161 ± 0.3	8.18 ± 0.26
H_2_O_2_/CoCl_2_-3m	−42 ± 3.0	90 ± 1.8	10.21 ± 0.61	160 ± 1.7	8.28 ± 0.34
NaOH-2w	−42 ± 0.6	90 ± 0.4	10.44 ± 0.63	160 ± 1.0	9.14 ± 0.56
NaOH-1m	−42 ± 1.0	89 ± 1.7	8.74 ± 0.09	159 ± 0.7	7.23 ± 0.07
NaOH-3m	−42 ± 3.8	87 ± 2.5	6.07 ± 1.17	150 ± 4.2	6.05 ± 0.49

**Table 2:** Impact of ageing under accelerated conditions on Mw, Mn and Ð values (mean ± SD). Molar masses are expressed in polystyrene equivalent.

	Mn (g/mol)	Mw (g/mol)	Ð
Control	25,600 ± 540	60,000 ± 2750	2.36 ± 0.06
H_2_O_2_/CoCl_2_-2w	26,000 ± 700	59,000 ± 1800	2.28 ± 0.08
H_2_O_2_/CoCl_2_-1m	24,900 ± 1100	59,100 ± 1800	2.37 ± 0.05
H_2_O_2_/CoCl_2_-3m	24,900 ± 920	58,600 ± 1700	2.36 ± 0.03
NaOH-2w	25,300 ± 850	60,000 ± 1650	2.37 ± 0.07
NaOH-1m	25,700 ± 560	60,800 ± 1250	2.37 ± 0.02
NaOH-3m	25,800 ± 320	61,100 ± 1100	2.36 ± 0.02

**Table 3:** Evolution of roughness (Ra) during ageing (*n* = 3).

	Control	2 Weeks	1 Months	3 Months
H_2_O_2_/CoCl_2_	11.0 ± 2.5	16.2 ± 1.9	16.2 ± 1.4	18.6 ± 0.5
NaOH		13.7 ± 1.8	14.6 ± 0.6	22.1 ± 1.4

**Table 4:** Young’s modulus (E), ultimate tensile strength (σ_max_), and strain at break (A) from tensile curves (mean ± SD) of control samples and aged one and three months in oxidative and hydrolytic conditions.

Samples	E (MPa)		σ_max_ (MPa)		A (%)	
	Para	Perp	Para	Perp	Para	Perp
TPU	13.8 ± 0.5	13.1 ± 0.6	11.0 ± 0.6	4.2 ± 0.2	752 ± 87	130 ± 33
H_2_O_2_/CoCl_2_-1m	12.8 ± 1.0	11.8 ± 0.5	9.8 ± 1.5	4.0 ± 0.3	720 ± 62	82 ± 42
H_2_O_2_/CoCl_2_-3m	13.2 ± 0.3	12.6 ± 0.4	10.3 ± 0.2	3.4 ± 0.8	746 ± 77	43 ± 22
NaOH-1m	12.9 ± 0.7	12.7 ± 1.2	9.9 ± 0.5	3.4 ± 0.8	778 ± 130	53 ± 13
NaOH-3m	12.8 ± 1.1	12.0 ± 2.3	10.6 ± 0.7	4.4 ± 0.5	801 ± 62	63 ± 35

## Data Availability

Data available on request due to restrictions.

## Supplementary Materials

The following supporting information can be downloaded at: https://www.mdpi.com/article/10.3390/ma18214948/s1, Figure S1: ATR-FTIR spectra of TPU and aged samples from 400 to 4000 cm^−1^; Figure S2: SEM images of the evolution of the EBS migration on aged sample in (a–c) oxidizing and (d,e) hydrolytic conditions; Figure S3: SEM images of unaged TPU.

## Abbreviations

The following abbreviations are used in this manuscript:

A	Strain at break
Ð	Polydispersity Index
DSC	Differential Scanning Calorimetry
E	Young’s Modulus
EBS	Ethylene-Bis-Stearamid
FDM	Fused Deposition Modelling
FTIR	Fourier Transform Infrared Spectroscopy
H_2_O_2_/CoCl_2_	20% H_2_O_2_/0.1 M CoCl_2_
HS	Hard Segments
Mn	Number average molecular weight
Mw	Weight average molecular weight
Ra	Roughness Average
ROS	Reactive Oxygen Species
SEC	Size Exclusion Chromatography
SEM	Scanning Electron Microscope
SS	Soft Segments
Tc	Crystallisation Temperature
Tg	Glass Transition Temperature
Tm	Melting Temperature
THF	TetraHydroFuran
TPU	Thermoplastic Polyurethane
ΔHc	Crystallisation Enthalpy
ΔHm	Melting Enthalpy
σ_max_	Ultimate Tensile Strength

## References

[1] Bajsić E.G., Šmit I., Leskovac M. (2007). Blends of Thermoplastic Polyurethane and Polypropylene. I. Mechanical and Phase Behavior. J. Appl. Polym. Sci..

[2] Drożdż K., Gołda-Cępa M., Brzychczy-Włoch M. (2024). Polyurethanes as Biomaterials in Medicine: Advanced Applications, Infection Challenges, and Innovative Surface Modification Methods. Adv. Microbiol..

[3] Grzęda D., Węgrzyk G., Nowak A., Komorowska G., Szczepkowski L., Ryszkowska J. (2023). Effect of Different Amine Catalysts on the Thermomechanical and Cytotoxic Properties of ‘Visco’-Type Polyurethane Foam for Biomedical Applications. Materials.

[4] Acierno D., Graziosi L., Patti A. (2023). Puncture Resistance and UV Aging of Nanoparticle-Loaded Waterborne Polyurethane-Coated Polyester Textiles. Materials.

[5] Jung Y.-S., Lee S., Park J., Shin E.-J. (2023). Synthesis of Novel Shape Memory Thermoplastic Polyurethanes (SMTPUs) from Bio-Based Materials for Application in 3D/4D Printing Filaments. Materials.

[6] He Y., Xie D., Zhang X. (2014). The Structure, Microphase-Separated Morphology, and Property of Polyurethanes and Polyureas. J. Mater. Sci..

[7] Petrović Z.S., Ferguson J. (1991). Polyurethane Elastomers. Prog. Polym. Sci..

[8] Cauich-Rodríguez J.V., Chan-Chan L.H., Hernandez-Sánchez F., Cervantes-Uc J.M., Pignatello R. (2013). Degradation of Polyurethanes for Cardiovascular Applications. Advances in Biomaterials Science and Biomedical Applications.

[9] Ghanbari H., Viatge H., Kidane A.G., Burriesci G., Tavakoli M., Seifalian A.M. (2009). Polymeric Heart Valves: New Materials, Emerging Hopes. Trends Biotechnol..

[10] Xie F., Zhang T., Bryant P., Kurusingal V., Colwell J.M., Laycock B. (2019). Degradation and Stabilization of Polyurethane Elastomers. Prog. Polym. Sci..

[11] Stokes K., McVenes R., Anderson J.M. (1995). Polyurethane Elastomer Biostability. J. Biomater. Appl..

[12] Hung K.-C., Tseng C.-S., Hsu S.-H., Cooper S.L., Guan J. (2016). 5–3D Printing of Polyurethane Biomaterials. Advances in Polyurethane Biomaterials.

[13] Hua W., Shi W., Mitchell K., Raymond L., Coulter R., Zhao D., Jin Y. (2022). 3D Printing of Biodegradable Polymer Vascular Stents: A Review. Chin. J. Mech. Eng. Addit. Manuf. Front..

[14] Pavan Kalyan B.G., Kumar L. (2022). 3D Printing: Applications in Tissue Engineering, Medical Devices, and Drug Delivery. AAPS PharmSciTech.

[15] Laverne F., Segonds F., Dubois P. (2016). Fabrication additive–Principes généraux. Tech. L’ingénieur.

[16] Lee S.J., Jo H.H., Lim K.S., Lim D., Lee S., Lee J.H., Kim W.D., Jeong M.H., Lim J.Y., Kwon I.K. (2019). Heparin Coating on 3D Printed Poly (l-Lactic Acid) Biodegradable Cardiovascular Stent via Mild Surface Modification Approach for Coronary Artery Implantation. Chem. Eng. J..

[17] Mirasadi K., Yousefi M.A., Jin L., Rahmatabadi D., Baniassadi M., Liao W.-H., Bodaghi M., Baghani M. (2025). 4D Printing of Magnetically Responsive Shape Memory Polymers: Toward Sustainable Solutions in Soft Robotics, Wearables, and Biomedical Devices. Adv. Sci..

[18] M’Bengue M.-S., Mesnard T., Chai F., Maton M., Gaucher V., Tabary N., García-Fernandez M.-J., Sobocinski J., Martel B., Blanchemain N. (2023). Evaluation of a Medical Grade Thermoplastic Polyurethane for the Manufacture of an Implantable Medical Device: The Impact of FDM 3D-Printing and Gamma Sterilization. Pharmaceutics.

[19] Desai S.M., Sonawane R.Y., More A.P. (2023). Thermoplastic Polyurethane for Three-Dimensional Printing Applications: A Review. Polym. Adv. Technol..

[20] Guerra A.J., Cano P., Rabionet M., Puig T., Ciurana J. (2018). 3D-Printed PCL/PLA Composite Stents: Towards a New Solution to Cardiovascular Problems. Materials.

[21] Wei J., Oyunbaatar N.-E., Jeong Y.-J., Park J., Kim S.-H., Kwon K., Lee H., Won Y., Kim D.-S., Lee D.-W. (2025). Enhancing Flexibility of Smart Bioresorbable Vascular Scaffolds through 3D Printing Using Polycaprolactone and Polylactic Acid. Sens. Actuators B: Chem..

[22] Zhai Y., Sun Z., Zhang T., Zhou C., Kong X. (2024). Mechanical Property of Thermoplastic Polyurethane Vascular Stents Fabricated by Fused Filament Fabrication. Micromachines.

[23] Martin N.K., Domínguez-Robles J., Stewart S.A., Cornelius V.A., Anjani Q.K., Utomo E., García-Romero I., Donnelly R.F., Margariti A., Lamprou D.A. (2021). Fused Deposition Modelling for the Development of Drug Loaded Cardiovascular Prosthesis. Int. J. Pharm..

[24] Walker T.G., Kalva S.P., Yeddula K., Wicky S., Kundu S., Drescher P., d’Othee B.J., Rose S.C., Cardella J.F. (2010). Clinical Practice Guidelines for Endovascular Abdominal Aortic Aneurysm Repair: Written by the Standards of Practice Committee for the Society of Interventional Radiology and Endorsed by the Cardiovascular and Interventional Radiological Society of Europe and the Canadian Interventional Radiology Association. J. Vasc. Interv. Radiol..

[25] Fanelli F., Dake M.D. (2009). Standard of Practice for the Endovascular Treatment of Thoracic Aortic Aneurysms and Type B Dissections. Cardiovasc. Interv. Radiol..

[26] Blanchemain N., Martel B., Tabary N., Garcia Fernandez M.-J., M’bengue M.-S., Mesnard T., Sobocinski J., Hildebrand F. Fabrication D’une Endoprothèse Par Impression 3d. https://patentscope.wipo.int/search/fr/WO2024213841.

[27] Anderson J.M., Hiltner A., Wiggins M.J., Schubert M.A., Collier T.O., Kao W.J., Mathur A.B. (1998). Recent Advances in Biomedical Polyurethane Biostability and Biodegradation. Polym. Int..

[28] Christenson E.M., Anderson J.M., Hiltner A. (2007). Biodegradation Mechanisms of Polyurethane Elastomers. Corros. Eng. Sci. Technol..

[29] Davies P., Evrard G. (2007). Accelerated Ageing of Polyurethanes for Marine Applications. Polym. Degrad. Stab..

[30] Christenson E.M., Anderson J.M., Hiltner A. (2004). Oxidative Mechanisms of Poly(Carbonate Urethane) and Poly(Ether Urethane) Biodegradation:In Vivo and in Vitro Correlations. J. Biomed. Mater. Res..

[31] Christenson E.M., Dadsetan M., Wiggins M., Anderson J.M., Hiltner A. (2004). Poly(Carbonate Urethane) and Poly(Ether Urethane) Biodegradation: In Vivo Studies. J. Biomed. Mater. Res..

[32] Drobny J.G. (2014). Thermoplastic Polyurethane Elastomers. Handbook of Thermoplastic Elastomers.

[33] Feng Y., Li C. (2006). Study on Oxidative Degradation Behaviors of Polyesterurethane Network. Polym. Degrad. Stab..

[34] Christenson E.M., Patel S., Anderson J.M., Hiltner A. (2006). Enzymatic Degradation of Poly(Ether Urethane) and Poly(Carbonate Urethane) by Cholesterol Esterase. Biomaterials.

[35] Weems A.C., Wacker K.T., Carrow J.K., Boyle A.J., Maitland D.J. (2017). Shape Memory Polyurethanes with Oxidation-Induced Degradation: In Vivo and in Vitro Correlations for Endovascular Material Applications. Acta Biomater..

[36] Chaffin K.A., Chen X., McNamara L., Bates F.S., Hillmyer M.A. (2014). Polyether Urethane Hydrolytic Stability after Exposure to Deoxygenated Water. Macromolecules.

[37] (2013). Stérilisation des Produits de Santé—Irradiation—Partie 2: Établissement de la Dose Stérilisante.

[38] (2013). Évaluation Biologique des Dispositifs Médicaux—Partie 13: Identification et Quantification de Produits de Dégradation de Dispositifs Médicaux à Base de Polymères.

[39] (2021). Spécification Géométrique des Produits (GPS)—État de Surface: Surfacique Partie 2: Termes, Définitions et Paramètres D’états de Surface.

[40] (2022). Standard Test Method for Tensile Properties of Plastics.

[41] Król P., Pilch-Pitera B. (2007). Phase Structure and Thermal Stability of Crosslinked Polyurethane Elastomers Based on Well-Defined Prepolymers. J. Appl. Polym. Sci..

[42] Schollenberger C.S., Stewart F.D. (1971). Thermoplastic Polyurethane Hydrolysis Stability. J. Elastoplast..

[43] Mrad O., Saunier J., Aymes Chodur C., Rosilio V., Agnely F., Aubert P., Vigneron J., Etcheberry A., Yagoubi N. (2010). A Comparison of Plasma and Electron Beam-Sterilization of PU Catheters. Radiat. Phys. Chem..

[44] Mrad O., Saunier J., Aymes-Chodur C., Mazel V., Rosilio V., Agnely F., Vigneron J., Etcheberry A., Yagoubi N. (2011). Aging of a Medical Device Surface Following Cold Plasma Treatment: Influence of Low Molecular Weight Compounds on Surface Recovery. Eur. Polym. J..

[45] Nouman M., Saunier J., Jubeli E., Marlière C., Yagoubi N. (2017). Impact of Sterilization and Oxidation Processes on the Additive Blooming Observed on the Surface of Polyurethane. Eur. Polym. J..

[46] Christenson E.M., Anderson J.M., Hiltner A. (2006). Antioxidant Inhibition of Poly(Carbonate Urethane)in Vivo Biodegradation. J. Biomed. Mater. Res..

[47] Nouman M., Saunier J., Jubeli E., Yagoubi N. (2017). Additive Blooming in Polymer Materials: Consequences in the Pharmaceutical and Medical Field. Polym. Degrad. Stab..

[48] Nouman M., Jubeli E., Saunier J., Yagoubi N. (2016). Exudation of Additives to the Surface of Medical Devices: Impact on Biocompatibility in the Case of Polyurethane Used in Implantable Catheters. J. Biomed. Mater. Res. Part. A.

[49] Ludwick A., Aglan H., Abdalla M.O., Calhoun M. (2008). Degradation Behavior of an Ultraviolet and Hygrothermally Aged Polyurethane Elastomer: Fourier Transform Infrared and Differential Scanning Calorimetry Studies. J. Appl. Polym. Sci..

[50] Jiao L., Xiao H., Wang Q., Sun J. (2013). Thermal Degradation Characteristics of Rigid Polyurethane Foam and the Volatile Products Analysis with TG-FTIR-MS. Polym. Degrad. Stab..

[51] Jayaraman A., Kang J., Antaki J.F., Kirby B.J. (2023). The Roles of Sub-Micron and Microscale Roughness on Shear-Driven Thrombosis on Titanium Alloy Surfaces. Artif. Organs.

[52] Hecker J.F., Scandrett L.A. (1985). Roughness and Thrombogenicity of the Outer Surfaces of Intravascular Catheters. J. Biomed. Mater. Res..

[53] Linneweber J., Dohmen P.M., Kerzscher U., Affeld K., Nosé Y., Konertz W. (2007). The Effect of Surface Roughness on Activation of the Coagulation System and Platelet Adhesion in Rotary Blood Pumps. Artif. Organs.

