# Supplementary material for: In Vitro Degradation Studies of 3D-Printed Thermoplastic Polyurethane for the Design of Vascular Implant
Source: Materials (Basel). 2025 Oct 29;18(21):4948. doi: 10.3390/ma18214948 (PMC12608279; doi:10.3390/ma18214948)
Supplement: Supplementary file 1 [file materials-18-04948-s001.zip › materials-3935418-supplementary.pdf]

Supplementary data

In vitro degradation studies of 3D-printed thermoplastic polyurethane for vascular implant applications

Kim Vanden Broeck[1,2], Marie-Stella M'Bengue[1,2], Thomas Mesnard[1,3], Mickaël Maton[1], Nicolas Tabary[2], Jonathan Sobocinski[1,3], Bernard Martel[2], Nicolas Blanchemain[1]

[1]Univ. Lille, INSERM, CHU Lille, U1008 – ADDS, F-59000 Lille, France
[2]Univ. Lille, CNRS, INRAE, Centrale Lille, UMR 8207—UMET—Unité Matériaux et Transformations, F-59000 Lille, France
[3]Institut Coeur Poumon, Regional Hospital Center University of Lille (CHRU Lille), 2 Avenue Oscar Lambret, F-59000 Lille, France

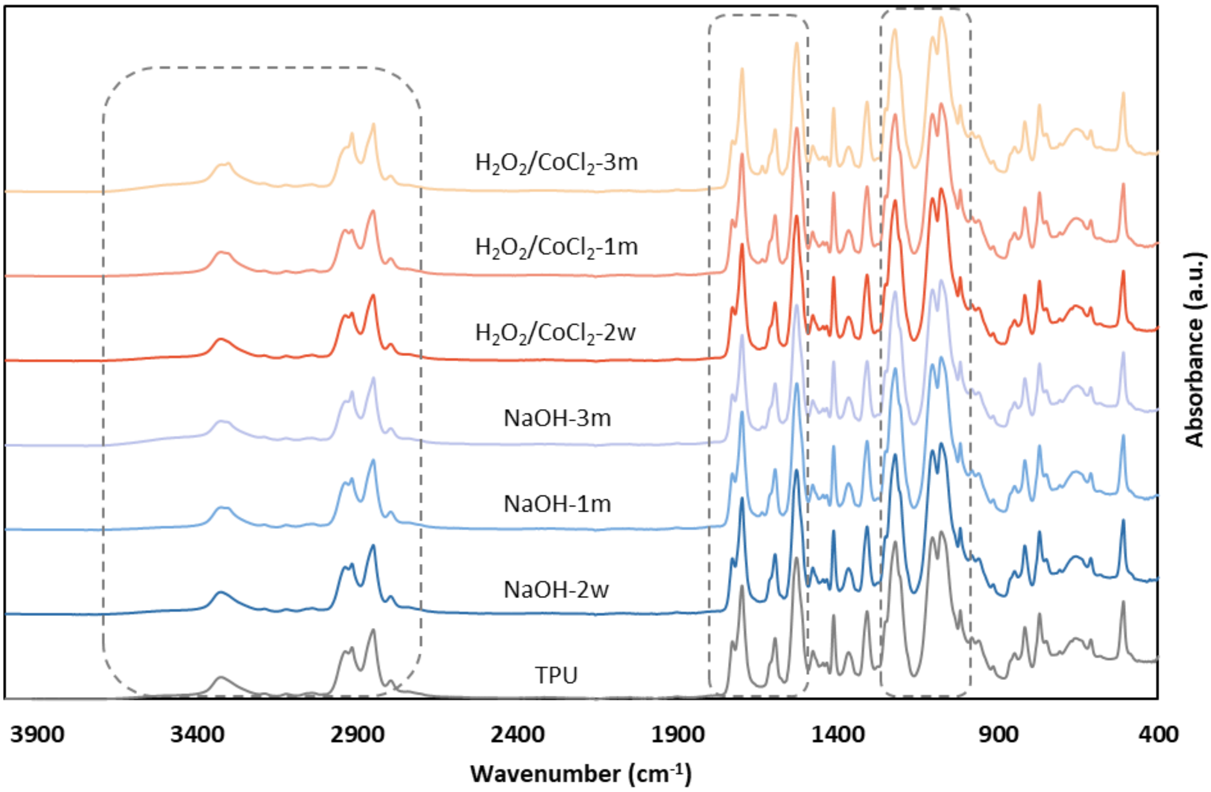

Figure S1. ATR-FTIR spectra of PTU and aged samples from 400 to 4000 cm^{-1}

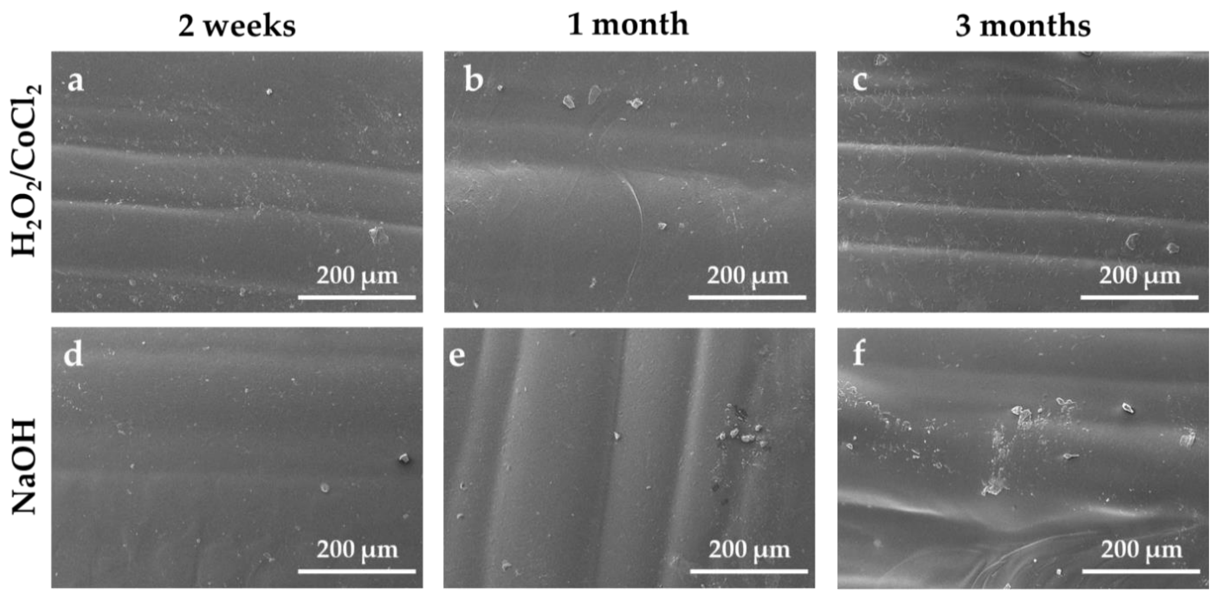

Figure S2. SEM images of the evolution of the EBS migration on aged sample in (a-c) oxidizing and (d-e) hydrolytic conditions

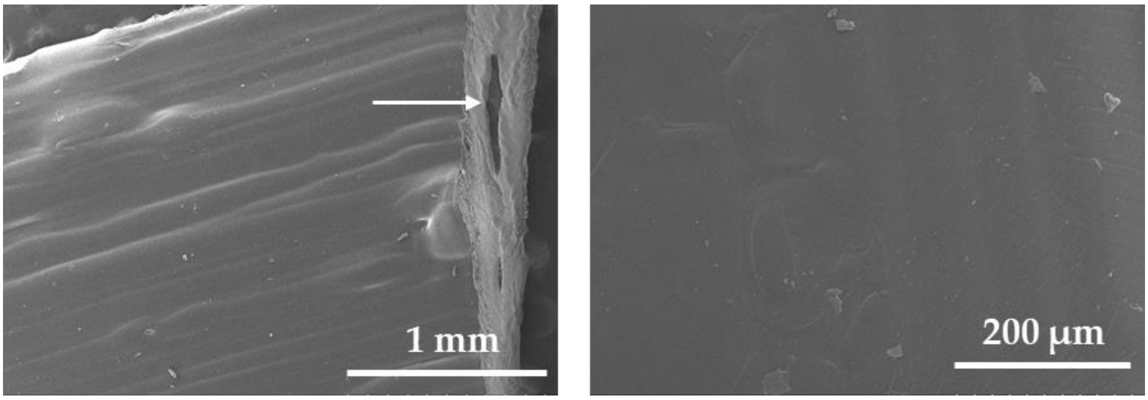

Figure S3. SEM images of unaged TPU
